# The association of polyunsaturated fatty acids and asthma: a cross-sectional study

**DOI:** 10.1186/s41043-023-00435-w

**Published:** 2023-09-01

**Authors:** Gangtie Liu, Hengbo Ye, Qian Cheng, Jian Zhao, Congcong Ma, Huichao Jie

**Affiliations:** Department of Pediatrics, Taian Maternal and Child Healthcare Hospital, No.386 Longtan Road, Taian, 271000 Shandong Province People’s Republic of China

**Keywords:** Polyunsaturated fatty acids, Asthma, Children, NHANES database, Docosahexaenoic

## Abstract

**Background:**

To examine the relationships between polyunsaturated fatty acids (PUFAs) dietary intake and asthma in children.

**Methods:**

In this cross-sectional study, a total of 14,727 participants from the United States National Health and Nutrition Examination Survey (NHANES) database in 1999–2018 were included, and the baseline characteristics of all participants were gathered. The description analysis was used to explore the possible covariates. Weighted multivariate logistic regression models were adopted to assessed the association between PUFAs dietary intake and asthma in children. In addition, we also performed subgroup analysis based on gender, age, and maternal smoking during pregnancy to investigate this relationship.

**Results:**

The prevalence of asthma approximately was 15.38% in the present study. The result of weighted multivariate logistic regression indicated that, docosahexaenoic [weighted odds ratio (OR) = 0.37, 95% confidence interval (CI) 0.19–0.74], total *n* − 3 PUFAs (weighted OR = 0.63, 95%CI 0.43–0.91), and eicosapentaenoic (weighted OR = 0.35, 95%CI 0.13–0.95) dietary intake were negatively associated with asthma in children. The subgroup analysis described that when children were male (weighted OR = 0.28, 95%CI 0.10–0.84), or were 5–7 years (weighted OR = 0.04, 95%CI 0.01–0.37), were 7–12 years (weighted OR = 0.46, 95%CI 0.24–0.90), or their maternal smoking during pregnancy (weighted OR = 0.16, 95%CI 0.03–0.90), docosahexaenoic dietary intake was negatively related to childhood asthma.

**Conclusion:**

Docosahexaenoic dietary intake was negatively associated with the asthma in children, especially if children were male, or were 5–12 years, or their maternal smoking during pregnancy.

## Background

Asthma, as a type of chronic airway inflammation, is widely recognized as the most prevalent pulmonary disease among children [1]. While the overall prevalence of asthma remained relatively stable, recent statistics indicate that approximately one in 20 children suffer from asthma worldwide [2], and the prevalence of childhood asthma in US about 6.5% [3], which continues to impose an increasing burden on healthcare systems.

Given evidence have reported that genetic, environmental, and behavioral changes are associated with the development of childhood asthma, particularly in relation to dietary factors [4, 5]. Garcia-Larsen et al. have pointed out that some components of foods have antioxidant, anti-allergic, and anti-inflammatory properties, which could play a beneficial role in preventing the risk of asthma [5]. Polyunsaturated fatty acids (PUFAs) including *n* − 3 and *n* − 6 fatty acids were an essential nutrient found in many foods, including fish, fruits, soybean oils, purslane, and nuts [6]. Previous research has demonstrated the significant role of PUFAs in energy storage and transportation, metabolism, gene regulation, as well as asthma risk [7, 8]. A systematic review has concluded that *n* − 3 PUFAs (primarily docosahexaenoic acid and eicosapentaenoic acid) have a beneficial effect on asthma, possibly due to their ability to resolve inflammation [7]. Additionally, a prospective cohort study conducted on young adults in the United States revealed a negative longitudinal association between the intake of total *n* − 3 PUFA, docosahexaenoic acid, eicosapentaenoic acid, respectively, and the incidence of asthma [9]. Nevertheless, as far as we know, current studies have focused on the association between PUFAs intake and asthma among adults, with little attention paid to the effect in children. Recently, a cross-sectional analysis has shown that the intake of total *n* − 3 and total *n* − 6 PUFAs is inversely associated with the risk of asthma among 3-year-old children [10], but this study had limitations due to its small sample size (*n* = 738) and focus on only 3-year-old children. For children older than 3 years, the association between PUFA and childhood asthma risk remains unclear.

In view of the lack of epidemiological information regarding the relationship of PUFA intake and asthma in children, we designed a cross-sectional study in an attempt to examine the relationship between PUFA's dietary intake and asthma in children aged 2–12 years, which provided a reference for dietary management of childhood asthma.

## Methods

### Data sources and study eligibility criteria

All participants of this cross-sectional study derived from the United States National Health and Nutrition Examination Survey (NHANES) database. NHANES is a nationally representative survey that employs a complex, stratified multistage probability sampling design [11]. The NHANES interview includes demographic, socioeconomic, dietary, and health-related questions and were conducted in the homes of the respondents. The examination component, consists of medical, dental, physiological measurements, and laboratory tests, was conducted in specially designed and equipped mobile centers. [12].

A total of 115,061 participants from the NHANES database in 1999–2018 were included in this cross-sectional study. We involved patients who were aged with 2–12 years old and had the information of asthma. Some participants who had missing information of mother's age, weight at birth, maternal smoking during pregnancy, family poverty income ratio (PIR), body mass index (BMI), and total fat intake were excluded. Only publicly available data were obtained in the present study, and the Ethics Committee of Taian Maternal and Child Healthcare Hospital exempted from the requirement of the ethical review. All methods of the study were conducted in line with the Declaration of Helsinki.

### Data collection

The data of all participants were gathered as following: age (years), gender, race, standing height (cm), weight (kg), BMI (kg/m^2^), mother's age (years), weight at birth (pounds), maternal smoking during pregnancy, family PIR, cotinine (ng/mL), energy (kcal), total fat (gm), PUFAs, and asthma.

### Polyunsaturated fatty acid measurement

The dietary interview component, known as What We Eat in America (WWEIA), was conducted face-to-face at a mobile examination center (MEC). The MEC diet interview room is equipped with a comprehensive set of measurement tools, including various types of glassware, bowls, cups, household spoons, measuring cups and spoons, rulers, thickness gauges, bean bags, and circles. These guidelines facilitate participants in accurately reporting the quantities of food they consume (https://wwwn.cdc.gov/Nchs/Nhanes/2003-2004/DR1TOT_C.htm#DR1TCARB) PUFAs intakes were calculated based on the U.S. Department of Agriculture’s Dietary Study Food and Nutrition Database for Dietary Studies [13]. In this study, PUFAs included *n* − 3 PUFAs and *n* − 6 PUFAs. Of which, *n* − 3 PUFAs included octadecatrienoic acid (gm), octadecatetraenoic acid (gm), eicosapentaenoic acid (gm), docosapentaenoic (gm), docosahexaenoic acid (gm). *n* − 6 PUFAs included octadecadienoic acid (gm), and eicosatetraenoic acid (gm). *n* − 3 PUFA's and *n* − 6 PUFA's intakes were obtained from the first day dietary recall data.

### Asthma assessment

The primary outcome of the analysis was defined as the occurrence of asthma. Asthma diagnosis was determined by the question “Has a doctor or other health professional ever told you that you have asthma?,” and participants who answered “Yes” were considered to have asthma.

### Statistical analysis

Considering the complex sampling design of the NHANES database, we conducted a weighted analysis. The measurement data of normal distribution were described as the weighted mean ± standard deviation (Mean ± SD), and group comparisons were conducted using *t*-test. The weighted median and quartile spacing [M (Q1, Q3)] were used to express the measurement data of non-normal distribution, while the Mann–Whitney U rank-sum test was employed for intergroup comparison. The categorical data were presented as weighted number of cases and the composition ratio [*n* (%)], and group comparisons were conducted using *χ*^2^ test.

First of all, we performed the description analysis of baseline information in the study, which aimed to explore the possible covariables, which had effect on the outcome. Then, we interpreted the association between PUFA's dietary intake and asthma in children by using weighted multivariate logistic regression models. Covariate adjustments: Model 1 adjusted for energy; Model 2 adjusted for energy, total fat, maternal smoking during pregnancy, weight at birth, age, BMI, race, gender, and family PIR. In addition, the association between PUFA's dietary intake and asthma in children was assessed based on the gender, age, and maternal smoking during pregnancy population. SAS (version 9.4) software was used for statistical analyses. Weighted odds ratio (OR) and 95% confidence interval (CI) were calculated in the study. *P* < 0.05 was considered as statistically significant difference.

## Results

### Population characteristics

After excluded some subjects who were aged > 12 years (*n* = 70,113) and had the missing information of asthma (*n* = 18,323), mother's age (*n* = 2,361), weight at birth (*n* = 376), maternal smoking during pregnancy (*n* = 68), family PIR (*n* = 1,292), BMI (*n* = 4,544), and total fat intake (*n* = 3,257), a total of 14,727 eligible participants were included eventually. These eligible participants were divided into the asthma group (*n* = 2,265) and non-asthma group (*n* = 12,462). The prevalence of asthma approximately was 15.38% in this study. Characteristics of all eligible participants were shown in Table 1. Compared to children without asthma, children who developed asthma were more likely to be male, to have higher age, lower mother's age, higher standing height, higher weight, lower Family PIR, higher BMI, higher energy, and higher total fat (*P* < 0.05) (Table 1). The average total PUFA in the overall population was 13.70 [standard deviation (SD) = 8.16] gm, with an average of 1.16 (SD = 0.74) gm for octadecatrienoic acid, 0.01 (SD = 0.02) gm for octadecatetraenoic acid, 0.01 (SD = 0.05) gm for eicosapentaenoic acid, 0.01 (SD = 0.02) gm for docosapentaenoic acid, and 0.03 (SD = 0.08) gm for docosahexaenoic acid.

**Table 1:** Characteristics of all participants

Variables	Total (*n* = 14,727)	Asthma group (*n* = 2265)	Non-asthma group (*n* = 12,462)	*P*
Age, (years), mean (S.E)	7.11 (0.04)	7.86 (0.08)	6.98 (0.04)	< 0.001
Age, (years), *n* (%)				< 0.001
≥ 2 and < 5	4368 (26.28)	489 (17.22)	3879 (27.82)	
≥ 5 and < 7	2546 (17.85)	384 (16.89)	2162 (18.01)	
≥ 7 and ≤ 12	7813 (55.87)	1392 (65.89)	6421 (54.16)	
Gender, *n* (%)				< 0.001
Male	7384 (50.94)	1318 (58.67)	6066 (49.62)	
Female	7343 (49.06)	947 (41.33)	6396 (50.38)	
Race/ethnicity, *n* (%)				< 0.001
Mexican American	3831 (14.52)	410 (10.79)	3421 (15.16)	
Other Hispanic	1261 (6.64)	235 (8.06)	1026 (6.40)	
Non-Hispanic White	4431 (57.41)	601 (53.15)	3830 (58.13)	
Non-Hispanic Black	3891 (13.75)	804 (19.87)	3087 (12.70)	
Other	1313 (7.68)	215 (8.13)	1098 (7.60)	
Standing height (cm), Mean (S.E)	125.86 (0.25)	130.91 (0.57)	124.99 (0.27)	< 0.001
Weight (kg), mean (S.E)	30.53 (0.18)	34.83 (0.48)	29.79 (0.19)	< 0.001
BMI (kg/m^2^), mean (S.E)	18.07 (0.05)	19.14 (0.13)	17.88 (0.05)	< 0.001
Mother's age (years), mean (S.E)	27.38 (0.11)	26.90 (0.21)	27.46 (0.11)	0.007
Weight at birth (pounds), mean (S.E)	6.87 (0.02)	6.71 (0.04)	6.90 (0.02)	< 0.001
Maternal smoking during pregnancy, *n* (%)				< 0.001
Yes	1901 (14.76)	393 (18.99)	1508 (14.04)	
No	12,826 (85.24)	1872 (81.01)	10,954 (85.96)	
Family PIR, mean (S.E)	2.41 (0.04)	2.30 (0.06)	2.43 (0.04)	0.013
Cotinine (ng/mL), mean (S.E)	0.54 (0.04)	0.57 (0.05)	0.54 (0.04)	0.501
Energy (kcal), mean (S.E)	1827.97 (7.82)	1950.55 (23.47)	1807.09 (7.95)	< 0.001
Total fat (gm), Mean (S.E)	67.72 (0.39)	72.93 (1.02)	66.84 (0.41)	< 0.001

PIR, poverty income ratio; BMI, body mass index

### The association between PUFA's dietary intake and the odds of asthma in children

As shown in Table 2, the result of weighted multivariate logistic regression indicated that, after adjusting for energy, docosahexaenoic acid dietary intake was associated with the lower odds of asthma in children (Model 1: weighted OR = 0.46, 95%CI 0.23–0.90), with similar results in Model 2 (weighted OR = 0.37, 95%CI 0.19–0.74). Furthermore, we also found that total *n* − 3 PUFAs (Model 2: weighted OR = 0.63, 95%CI 0.43–0.91) and eicosapentaenoic acid (Model 2: weighted OR = 0.35, 95%CI 0.13–0.95) were related to the decreased odds of asthma in children after adjusting for energy, total fat, maternal smoking during pregnancy, weight at birth, age, BMI, race, gender, and family PIR. These results suggested that *n* − 3 PUFAs might be related to asthma.

**Table 2:** The association between PUFA's dietary intake and the odds of asthma in children

Variables	Model 1		Model 2	
	OR (95% CI)	*P*	OR (95% CI)	*P*
Total PUFAs (gm)	1.00 (0.99–1.01)	0.377	1.00 (0.99–1.01)	0.950
Total *n* − 3 PUFAs	0.73 (0.51–1.04)	0.084	0.63 (0.43–0.91)	0.016
Octadecatrienoic acid (gm)	0.93 (0.84–1.04)	0.235	0.87 (0.75–1.01)	0.079
Octadecatetraenoic acid (gm)	8.35 (0.92–75.60)	0.061	4.54 (0.45–45.94)	0.203
Eicosapentaenoic acid (gm)	0.46 (0.18–1.21)	0.119	0.35 (0.13–0.95)	0.042
Docosapentaenoic acid (gm)	3.39 (0.27–41.93)	0.343	0.66 (0.04–9.81)	0.761
Docosahexaenoic acid (gm)	0.46 (0.23–0.90)	0.026	0.37 (0.19–0.74)	0.006
Total *n* − 6 PUFAs	1.00 (0.99–1.01)	0.405	1.00 (0.90–1.10)	0.920
Octadecadienoic acid (gm)	1.01 (0.99–1.02)	0.327	1.05 (0.97–1.14)	0.255
Eicosatetraenoic acid (gm)	1.73 (0.93–3.23)	0.087	1.08 (0.56–2.09)	0.820
Total *n* − 3/total *n* − 6	0.06 (0.00–6.49)	0.241	0.02 (0.00–4.33)	0.161

OR, odds ratio; CI, confidence interval; polyunsaturated fatty acids, PUFAs;

Model 1 adjusted for energy;

Model 2 adjusted for energy, total fat, maternal smoking during pregnancy, weight at birth, age, BMI, race, gender, and family PIR

### Risk stratification based on gender, age, and maternal smoking during pregnancy

**Fig. 1:**
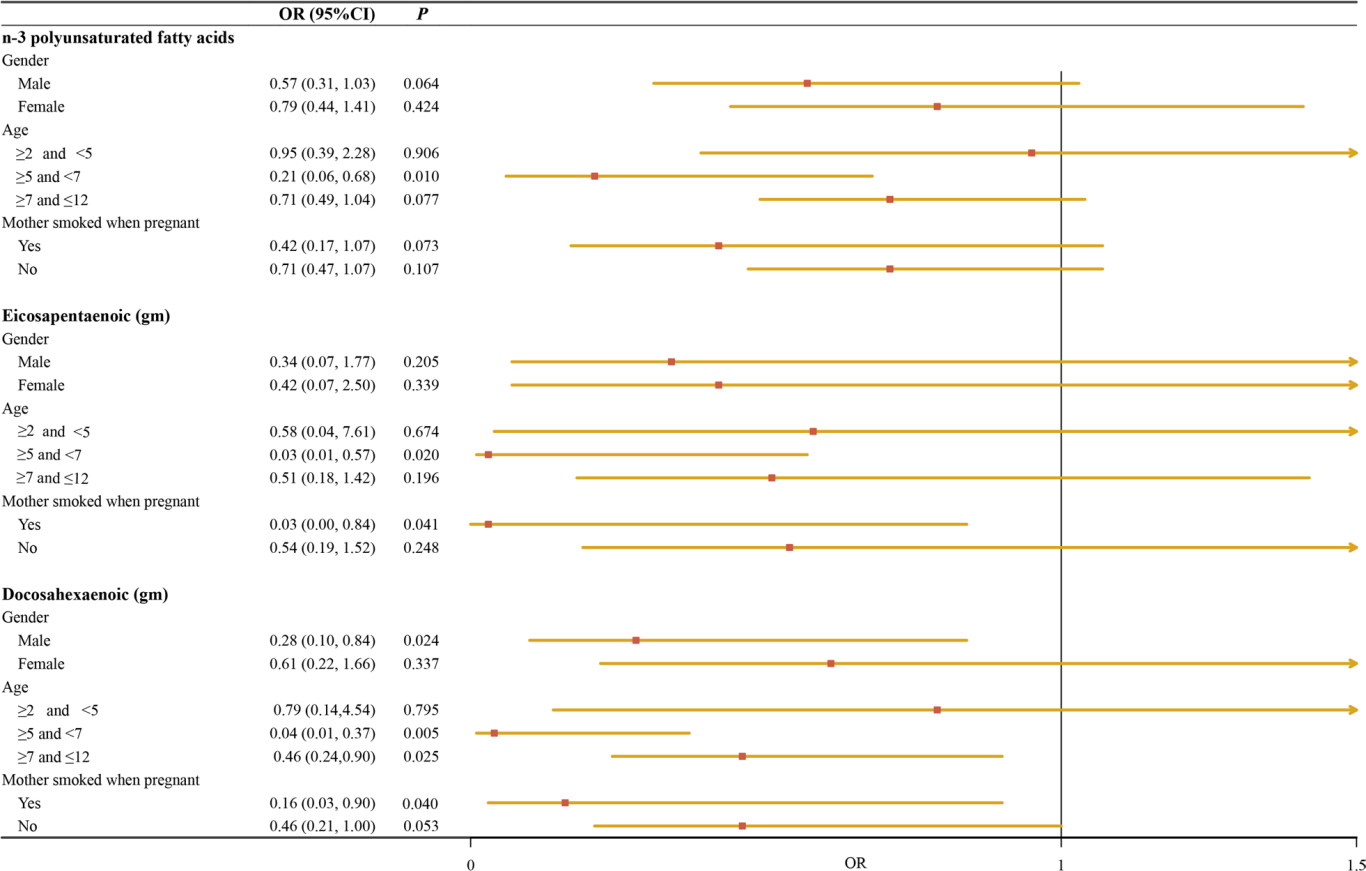
Subgroup analysis based on gender, age, and maternal smoking during pregnancy

We conducted a subgroup analysis based on the gender, age, and maternal smoking during pregnancy to explore the association between PUFA's dietary intake (total *n* − 3 PUFAs, docosahexaenoic acid and eicosapentaenoic acid) and the odds of asthma in children. The findings described (Fig. 1) that when children were male (weighted OR = 0.28, 95%CI 0.10–0.84), or were 7–12 years (weighted OR = 0.46, 95%CI 0.24–0.90), or their maternal smoking during pregnancy (weighted OR = 0.16, 95%CI 0.03–0.90), docosahexaenoic acid dietary intake was associated with a decreased odds of childhood asthma. Also, for children were 5–7 years, the associations of *n* − 3 PUFAs (weighted OR = 0.21, 95%CI 0.06–0.68), eicosapentaenoic acid (weighted OR = 0.03, 95%CI 0.01–0.57), and docosahexaenoic acid (weighted OR = 0.04, 95%CI 0.01–0.37) dietary intake on asthma were observed.

## Discussion

Currently, asthma remains a complex and heterogeneous chronic airway disease with high prevalence rates, particularly among children. [14]. Evidence from multiple studies suggested that dietary factors might play an important role in the risk of childhood asthma [15, 16]. However, there is a lack of evidence regarding the role of dietary intake of *n* − 3 and *n* − 6 PUFAs in childhood asthma development, and observational analyses have shown inconsistent associations between PUFAs and the odds of asthma in children [10, 17–19]. To better assess this association, we performed a cross-sectional analysis to investigate the relationship of *n* − 3 PUFAs, *n* − 6 PUFAs dietary intake and asthma among children aged 2–12 years old. The findings indicated that docosahexaenoic acid dietary intake, a type of *n* − 3 PUFAs, was associated with asthma in children, particularly among males or those aged 7–12 years old whose mothers smoked during pregnancy.

Generally, *n* − 6 PUFAs are considered to have proinflammatory effects, while *n* − 3 PUFAs have anti-inflammatory effects [20]. Not only that, several studies also have pointed out that *n* − 3 PUFAs possessed the most potent immunomodulatory activities among the fatty acids [21, 22]. In the current investigation, we have observed a potential association between dietary intake of docosahexaenoic acid and reduced odds of asthma in children. This result was consistent with previous study. In the study of Talaei, et al., they concluded that a higher intake of docosahexaenoic from fish during childhood was strongly associated with a lower risk of asthma in mid-adolescence [23]. The potential mechanism involves the modulation of inflammatory processes by docosahexaenoic acid through anti-inflammatory pathways, resulting in reduced odds of asthma. Lee-Sarwar, et al., showed that *n* − 3 PUFAs, *n* − 6 PUFAs intake was negatively associated with asthma in early childhood based on a relatively small sample size [10], which was inconsistent with the result of our study. Our study suggested that there was not significant difference between the *n* − 6 PUFAs intake and the odds of children asthma based on 14,727 population. The reason for the difference may be attributed to sample size.

When a mother smokes during pregnancy, it may induce epigenetic changes in the regulation of inflammatory genes, thereby increasing the risk of allergies in children [24]. We observed that the association of docosahexaenoic dietary and odds of asthma in children was significant in certain populations, including children were male, or were 5–7 years, or were 7–12 years or their maternal smoking during pregnancy. However, due to the current paucity of evidence, the exact mechanism underlying the association between dietary docosahexaenoic acid and asthma risk in different genders and maternal smoking remains unclear, this may be attributed to gender disparities [25] and the greater effect of maternal smoking on childhood asthma [24]. More research is needed in the future to explore these mechanisms. In addition, we also found that the average age of children in the non-asthma group was lower than that in the asthma group (Table 1). In this study, the diagnosis of asthma based on a single question may be applicable to individuals aged 6 years and older. Whereas some children may exhibit symptoms of asthma before the age of 5, this condition is often misdiagnosed or overlooked, which could explain why the non-asthma group tends to be younger in age.

As far as we know, this study is the first to investigate the association of PUFA's dietary intake and the odds of asthma among children based on the NHANES database. This study involved 14,727 children and implemented a subgroup analysis approach based on gender, age, and maternal smoking during pregnancy to further investigate the relationship between dietary intake of PUFAs and the odds of childhood asthma. Overall, these findings suggested that consuming a diet abundant in docosahexaenoic acid, such as fish, peanuts, walnuts, sesame seeds, and dried fruit, may have a beneficial impact on childhood asthma, particularly among males or children aged 5–12 or whose mothers engaged in smoking during pregnancy. Nevertheless, some study limitations also should be pointed: (1) we cannot determine a causal relationship between PUFA's dietary intake and the odds of children asthma in this cross-sectional study. We only found that docosahexaenoic acid dietary intake was related to the asthma in children; (2) the diagnosis of asthma in NHANES database relied on self-reported problems from the interview; therefore, the study lacks testing modalities on diagnosis, and the interviews may be subject to recall bias; (3) this data on PUFAs intake was the first day dietary recall interviews, which only reflects the short-term intake of participants and cannot account for the association of long-term dietary changes and asthma in children. The information on the assessment of the incorporation of the fatty acids into cell membranes is lacking in the database. Also, the influence of children's dietary patterns can be influenced by their parents, but this information was not available from the database; (4) some potential confounders might not have been accounted for in the study, such as use of asthma medications. More researches about the association should be conducted in the future.

## Conclusion

This study indicated that docosahexaenoic acid dietary intake may be negatively associated with asthma in children, especially if children were male, or were 5–12 years, or their maternal smoking during pregnancy. However, more prospective studies are still needed to confirm these findings.

## Data Availability

The datasets used and/or analyzed during the current study are available from the NHANES database, https://wwwn.cdc.gov/nchs/nhanes/.

## Abbreviations

PUFAs: Polyunsaturated fatty acids
NHANES: National Health and Nutrition Examination Survey
PIR: Poverty income ratio
BMI: Body mass index
Mean ± SD: Mean ± standard deviation
CI: Confidence interval
OR: Odds ratio

## References

[1] Pansare M, Seth D, Kamat D, Poowuttikul P (2021). Treatment for severe asthma in children: what about biologics?. Pediatr Ann.

[2] Asher MI, Rutter CE, Bissell K, Chiang CY, El Sony A, Ellwood E (2021). Worldwide trends in the burden of asthma symptoms in school-aged children: Global Asthma Network Phase I cross-sectional study. Lancet.

[3] Most Recent National Asthma Data|CDC [Internet]. 2020. [cited 2020 Sep 18]. https://www.cdc.gov/asthma/most_recent_national_asthma_data.htm

[4] Anderson HM, Jackson DJ (2017). Microbes, allergic sensitization, and the natural history of asthma. Curr Opin Allergy Clin Immunol.

[5] Garcia-Larsen V, Del Giacco SR, Moreira A, Bonini M, Charles D, Reeves T (2016). Asthma and dietary intake: an overview of systematic reviews. Allergy.

[6] Román GC, Jackson RE, Gadhia R, Román AN, Reis J (2019). Mediterranean diet: the role of long-chain ω-3 fatty acids in fish; polyphenols in fruits, vegetables, cereals, coffee, tea, cacao and wine; probiotics and vitamins in prevention of stroke, age-related cognitive decline, and Alzheimer disease. Rev Neurol (Paris).

[7] Kumar A, Mastana SS, Lindley MR (2016). n-3 Fatty acids and asthma. Nutr Res Rev.

[8] Wiktorowska-Owczarek A, Berezińska M, Nowak JZ (2015). PUFAs: structures, metabolism and functions. Adv Clin Exp Med.

[9] Li J, Xun P, Zamora D, Sood A, Liu K, Daviglus M (2013). Intakes of long-chain omega-3 (n-3) PUFAs and fish in relation to incidence of asthma among American young adults: the CARDIA study. Am J Clin Nutr.

[10] Lee-Sarwar K, Kelly RS, Lasky-Su J, Kachroo P, Zeiger RS, O'Connor GT (2019). Dietary and plasma polyunsaturated fatty acids are inversely associated with asthma and atopy in early childhood. J Allergy Clin Immunol Pract.

[11] Yang F, Liang H, Rosenthal RJ, Wexner SD (2021). The significant interaction between age and diabetes mellitus for colorectal cancer: evidence from NHANES data 1999–2016. Prim Care Diabetes.

[12] Kao CC, Yang ZY, Chen WL (2021). Association between protoporphyrin IX and sarcopenia: a cross sectional study. BMC Geriatr.

[13] Dong X, Li S, Chen J, Li Y, Wu Y, Zhang D (2020). Association of dietary ω-3 and ω-6 fatty acids intake with cognitive performance in older adults: National Health and nutrition examination Survey (NHANES) 2011–2014. Nutr J.

[14] Huang J, Pansare M (2019). New treatments for asthma. Pediatr Clin North Am.

[15] Saadeh D, Salameh P, Caillaud D, Charpin D, De Blay F, Kopferschmitt C (2015). Prevalence and association of asthma and allergic sensitization with dietary factors in schoolchildren: data from the french six cities study. BMC Public Health.

[16] Nwaru BI, Takkinen HM, Kaila M, Erkkola M, Ahonen S, Pekkanen J (2014). Food diversity in infancy and the risk of childhood asthma and allergies. J Allergy Clin Immunol.

[17] Mikkelsen A, Galli C, Eiben G, Ahrens W, Iacoviello L, Molnár D (2017). Blood fatty acid composition in relation to allergy in children aged 2–9 years: results from the European IDEFICS study. Eur J Clin Nutr.

[18] Miyake Y, Sasaki S, Arakawa M, Tanaka K, Murakami K, Ohya Y (2008). Fatty acid intake and asthma symptoms in Japanese children: the Ryukyus Child Health Study. Clin Exp Allergy.

[19] Murray CS, Simpson B, Kerry G, Woodcock A, Custovic A (2006). Dietary intake in sensitized children with recurrent wheeze and healthy controls: a nested case-control study. Allergy.

[20] Wendell SG, Baffi C, Holguin F (2014). Fatty acids, inflammation, and asthma. J Allergy Clin Immunol.

[21] D'Auria E, Miraglia Del Giudice M, Barberi S, Mandelli M, Verduci E, Leonardi S (2014). Omega-3 fatty acids and asthma in children. Allergy Asthma Proc.

[22] Simopoulos AP (2002). Omega-3 fatty acids in inflammation and autoimmune diseases. J Am Coll Nutr.

[23] Talaei M, Sdona E, Calder PC, Jones LR, Emmett PM, Granell R, et al. Intake of n-3 polyunsaturated fatty acids in childhood, FADS genotype and incident asthma. Eur Respir J. 2021; 58. 10.1183/13993003.03633-2020 PMC8411098 33509958

[24] Lee HS, Barraza-Villarreal A, Hernandez-Vargas H, Sly PD, Biessy C, Ramakrishnan U (2013). Modulation of DNA methylation states and infant immune system by dietary supplementation with ω-3 PUFA during pregnancy in an intervention study. Am J Clin Nutr.

[25] Extier A, Langelier B, Perruchot MH, Guesnet P, Van Veldhoven PP, Lavialle M (2010). Gender affects liver desaturase expression in a rat model of n-3 fatty acid repletion. J Nutr Biochem.

